# PET imaging of an optimized anti-PD-L1 probe ^68^Ga-NODAGA-BMS986192 in immunocompetent mice and non-human primates

**DOI:** 10.1186/s13550-022-00906-x

**Published:** 2022-06-13

**Authors:** Huimin Zhou, Guangfa Bao, Ziqiang Wang, Buchuan Zhang, Dan Li, Lixing Chen, Xiaoyun Deng, Bo Yu, Jun Zhao, Xiaohua Zhu

**Affiliations:** 1 Department of Nuclear Medicine, Tongji Hospital, Tongji Medical College, Huazhong University of Science and Technology, 1095 Jiefang Ave, Wuhan, 430030 China; 2 Department of Anatomy, School of Basic Medicine, Huazhong University of Science and Technology, Wuhan, 430030 Hubei Province China; 3 Cell Architecture Research Center, Huazhong University of Science and Technology, Wuhan, 430030 Hubei Province China

**Keywords:** PET imaging, ^68^Ga, Adnectin, BMS-986192, PD-L1, Cynomolgus

## Abstract

**Background:**

Adnectin is a protein family derived from the 10th type III domain of human fibronectin (^10^Fn3) with high-affinity targeting capabilities. Positron emission tomography (PET) probes derived from anti-programmed death ligand-1 (PD-L1) Adnectins, including ^18^F- and ^68^Ga-labeled BMS-986192, are recently developed for the prediction of patient response to immune checkpoint blockade. The ^68^Ga-labeled BMS-986192, in particular, is an attractive probe for under-developed regions due to the broader availability of ^68^Ga. However, the pharmacokinetics and biocompatibility of ^68^Ga-labeled BMS-986192 are still unknown, especially in non-human primates, impeding its further clinical translation.

**Methods:**

We developed a variant of ^68^Ga-labeled BMS-986192 using 1,4,7-triazacyclononane,1-glutaric acid-4,7-acetic acid (NODAGA) as the radionuclide–chelator. The resultant probe, ^68^Ga-NODAGA-BMS986192, was evaluated in terms of targeting specificity using a bilateral mouse tumor model inoculated with wild-type B16F10 and B16F10 transduced with human PD-L1 (hPD-L1-B16F10). The dynamic biodistribution and radiation dosimetry of this probe were also investigated in non-human primate cynomolgus.

**Results:**

^68^Ga-NODAGA-BMS986192 was prepared with a radiochemical purity above 99%. PET imaging with ^68^Ga-NODAGA-BMS986192 efficiently delineated the hPD-L1-B16F10 tumor at 1 h post-injection. The PD-L1-targeting capability of this probe was further confirmed using in vivo blocking assay and ex vivo biodistribution studies. PET dynamic imaging in both mouse and cynomolgus models revealed a rapid clearance of the probe via the renal route, which corresponded to the low background signals of the PET images. The probe also exhibited a favorable radiation dosimetry profile with a total-body effective dose of 6.34E-03 mSv/MBq in male cynomolgus.

**Conclusions:**

^68^Ga-NODAGA-BMS986192 was a feasible and safe tool for the visualization of human PD-L1. Our study also provided valuable information on the potential of targeted PET imaging using Adnectin-based probes.

**Supplementary Information:**

The online version contains supplementary material available at 10.1186/s13550-022-00906-x.

## Introduction

Blockade of the programmed cell death-1/programmed death ligand-1 (PD1/PD-L1) has shown impressive efficacy for the immunotherapy of patients with cancer [1–3]. Overexpression of PD-L1 in tumor cells and tumor-infiltrating lymphocytes, not only correlated with poor disease outcomes in multiple human cancers [4], but also predicted a better response to anti-PD-1/PD-L1 blockade [5]. Immunohistochemistry, as the gold standard to analyze the expression of target proteins, is unable to capture the dynamic changes of PD-L1 expression during tumor progression or to provide an overview picture of PD-L1 expression in heterogenic tumors [6]. Positron emission tomography (PET), as a noninvasive, sensitive, and quantifiable imaging modality, allows for a longitudinal and repetitive assessment of the expression of immune checkpoint proteins on a whole-body scale [7]. Recently, PET imaging using radiolabeled PD-L1-targeting molecules such as monoclonal antibodies [8–10], domain antibodies [11], affibodies [12], nanobodies [13], Adnectins [14], and peptides [15], specifically detect PD-L1 expression in a noninvasive manner. Several antibody-based imaging probes have been validated in clinical trials for visualizing PD-L1 positive tumors. Especially, the clinical responses of ^89^Zr-atezolizumab in the patients were better correlated with pretreatment PET signal than with immunohistochemistry-based predictive biomarker [16]. However, the slow clearance rate of antibody agents often takes many days to clear sufficiently from non-target tissues and enable imaging of the target. Moreover, unlabeled antibodies were often preinjected over the radiotracer to prevent non-specific accumulation in liver and blood pool and achieve higher target-to-background ratio [8, 9, 16].

Derived from the 10th type III domain of human fibronectin (^10^Fn3), Adnectins have comparable binding affinities with whole antibodies, but are smaller in size and simpler in structure [17]. The small size (~ 10 KDa) enables a rapid delivery of Adnectin-based probes to their targets and a fast glomerular clearance of the circulating probes from blood pool, and consequently yields PET images with good contrast. The absence of cysteine and disulfide bonds in Adnectin also makes it possible to introduce an exogenous cysteine for a site-specific labeling of PET radionuclides.

Currently, an Adnectin (ADX_5322_A02)-based anti-human PD-L1 (hPD-L1) probe, ^18^F-labeled BMS-986192 has produced a high-contrast imaging of PD-L1 positive lesions, and confirmed the correlation between the tumor uptake of the probe with the PD-L1 expression in patients with advanced non-small cell lung carcinoma (NSCLC) [18–20]. Another ^68^Ga-labeled BMS-986192 also produced high-contrast images to differentiate hPD-L1-positive tumors from hPD-L1-negative tumors in NSG mice [21]. However, there are still knowledge gaps to fill before a successful clinical translation of ^68^Ga-BMS-986192, such as the unknown pharmacokinetics and biocompatibility in non-rodent species. In addition, compared to that of 1,4,7,10-tetraazacyclododecane-*N*,*N*',*N,N'*-tetraacetic acid (DOTA), the coordination cavity formed by 1,4,7-triazacyclononane-*N*-glutamic acid-*N*′,*N*″-diacetic acid (NODAGA) chelates ^68^Ga with a higher stability [22]. Herein, we optimized the ^68^Ga labeling of BMS-986192 using a bifunctional chelator (BFC) maleimide-NODA-GA, and evaluated the binding affinity and stability of the resultant probe, ^68^Ga-NODAGA-BMS986192. Mice bearing hPD-L1-B16F10 tumors and healthy male cynomolgus were used to test the feasibility of ^68^Ga-NODAGA-BMS986192 for the in vivo imaging of PD-L1 expression and its safety profile.

## Materials and methods

### Reagents and equipment

The customized anti-PD-L1 Adnectin ADX_5322_A02 was prepared by ChinaPeptides Co. Ltd (Shanghai, China). The sequencing report, protein expression, and sodium dodecyl sulfate–polyacrylamide gel electrophoresis (SDS–PAGE) analysis are shown in Additional file 1: Figs. S1, S2. Maleimide-NODA-GA was purchased from CheMatech (France), and high-purity hydrochloric acid from Merck (Darmstadt, Germany). Other chemicals were purchased from Sigma-Aldrich. The ^68^Ga/^68^Ge generator was purchased from ITG Isotope Technologies Garching Gmbh (Germany). PD-10 columns were purchased from GE Healthcare (Chicago, USA). Matrix-assisted laser desorption/ionization time-of-flight (MALDI-TOF) mass spectrometry was performed on a Microflex LT/LRF system (Bruker Daltonics, Billerica, MA, USA). High-performance liquid chromatography (HPLC) /size exclusive chromatography (SEC) was performed on a Waters e2695 system equipped with a Superdex 200 Increase 10/300 GL column (GE Healthcare). Instant thin-layer chromatography (iTLC) was performed on Eckert & Ziegler Mini-Scan/FC (Hopkinton, MA, USA). The samples for binding affinity and biodistribution were measured with a gamma counter (Wizard 2480, Perkin Elmer Instruments Inc, Connection, USA).

### Synthesis and quality evaluation of.^68^Ga-NODAGA-BMS986192

**Fig. 1:**
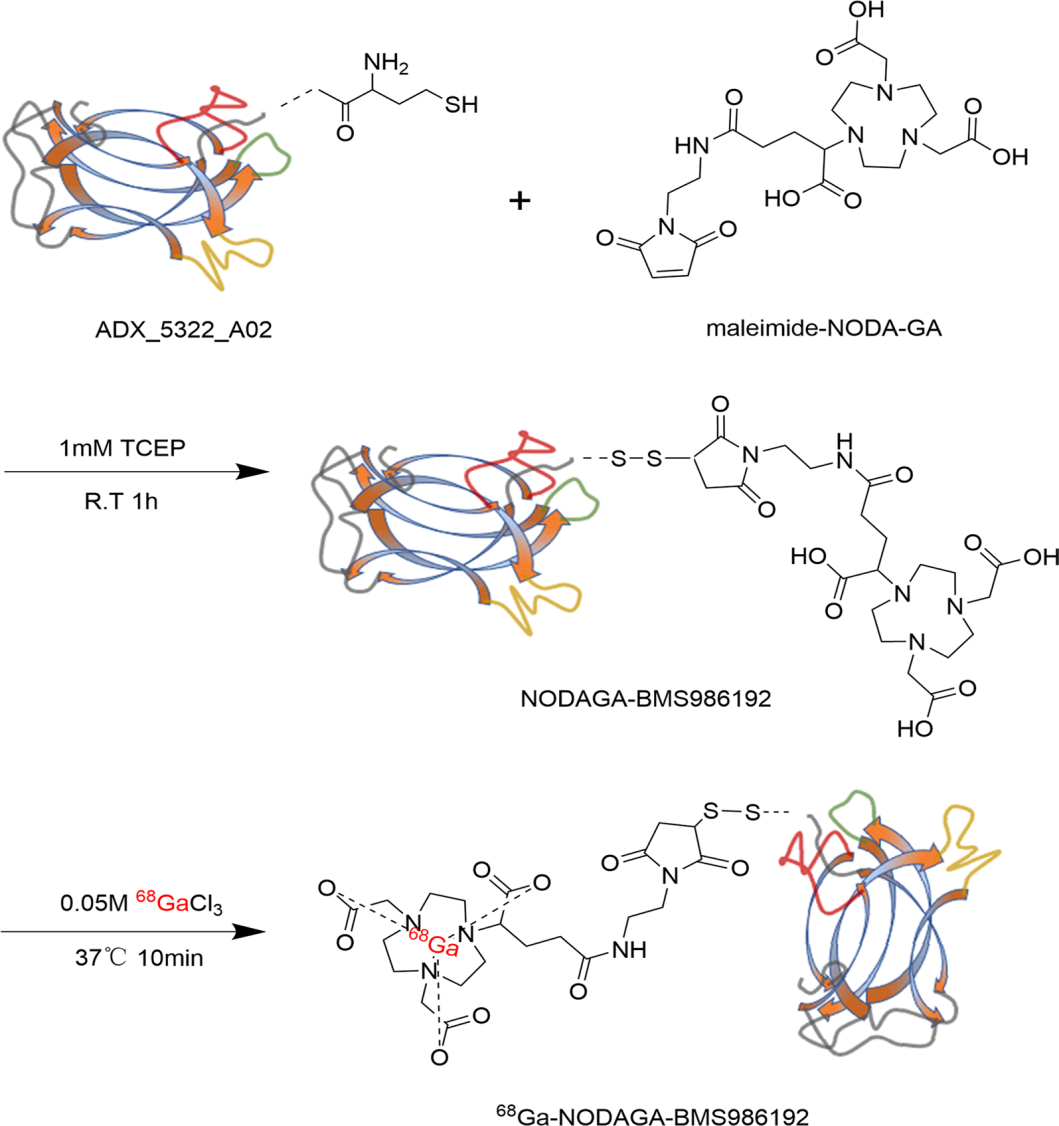
Synthesis schematic of ^68^Ga-NODAGA-BMS986192

The synthesis scheme of ^68^Ga-NODAGA-BMS986192 is shown in Fig. 1. Briefly, 50-fold molar excess of maleimide-NODA-GA was first dissolved in PBS (pH 7.4) and added to ADX_5322_A02 (0.2 mg) dissolved in 1 mM tris(2-chloroethyl) phosphate (TCEP) supplemented with 5% Dimethyl sulfoxide (DMSO) [23], followed by incubation at 25 °C for 1 h. The mixture was then purified and concentrated by centrifugal filtration to give NODAGA-conjugated ADX_5322_A02 (NODAGA-BMS986192). Radionuclide ^68^Ga (370 to 450 MBq) was eluted from a ^68^Ga/^68^Ge generator using 0.05 M HCl, mixed with the NODAGA-BMS986192 in sodium acetate, and then incubated at 37 °C for 10 min. The final product, ^68^Ga-NODAGA-BMS986192, was purified using a PD-10 column using saline as the eluent.

The radiochemistry yield (RCY) of ^68^Ga-NODAGA-BMS986192 was measured by the radio-iTLC. The radiochemistry purity (RCP) was measured by the radio-HPLC/SEC chromatography using 0.01 M phosphate buffer (pH 7.4) as the mobile phase at a flow rate of 1 mL/min. In vitro stability was tested by incubation in fresh human serum at 37 °C for 2 h. Cold ^69^GaCl_3_ solution was used for the synthesis of ^69^ Ga-NODAGA-BMS986192 as the standard reference.

### Cell lines and culture conditions

Human glioblastoma cell line LN-229 was purchased from BcNa Culture Collection Co. Ltd (Beijing, China). Mice melanoma cell lines B16-F10 and hPD-L1-gene transfected B16-F10 (hPD-L1-B16F10) were obtained from Ubigene Biosciences Co. Ltd (Guangzhou, China). The real-time qPCR results confirmed that the hCD274 (hPD-L1) RNA expression of hPD-L1-B16F10 is over 5000 times higher than that of the B16-F10 wild type (Additional file 1: Fig. S3). All cell lines were cultured in Dulbecco's Modified Eagle Medium (DMEM) with 10% fetal bovine serum (Gibco) and penicillin/streptomycin (100 U/mL), and incubated at 37℃ in a humidified incubator with 5% CO_2_.

### Binding affinity assay

Binding affinity was tested using a PD-L1 positive human glioblastoma cell line LN-229 [21]. Briefly, equal amount of ^68^Ga-labeled and unlabeled ADX_5322_A02 were mixed, and added to the cells at a total concentration ranging from 10^–11^ to 10^–6^ M. After 1 h incubation at 37 °C, cells were washed and centrifuged for 3 times with saline, all of the supernatants were collected as free radioligand. The amount of cell-bound activity (cell pellet) as well as the free radioligand was measured in a gamma counter (Wizard 2480, Perkin Elmer Instruments Inc, USA).

### Animal models

Bilateral mouse models were established by subcutaneous inoculation of hPD-L1-B16F10 cells (1 × 10^6^) and B16-F10 cells (1 × 10^6^) [24] at contralateral shoulders of 18–20 g female C57BL/6 mice at 5–7 weeks of age. PET images were acquired once the tumors grew to 500 to 750 mm^3^ in size [25]. Healthy male cynomolgus weighing 5–7 kg were obtained from TOPGENE BIOTECHNOLOGY Co. Ltd (Wuhan, China).

### PET imaging and ex vivo biodistribution in mice models

All scans were performed on an uBioEXPLORER PET/CT scanner (United Imaging Life Science Instruments, China). Mice (*n* = 4/group) were intravenously injected with 3.7–5.6 MBq (100–150 μci) ^68^Ga-NODAGA-BMS986192. Dynamic PET scans were recorded from 2 to 120 minutes post-injection. For blocking groups, cold ADX_5322_A02 (10 mg/kg) was simultaneously injected with ^68^Ga-NODAGA-BMS986192. Data were reconstructed in 16 time frames (10 × 2 min, 10 × 10 min) using an ordered subsets expectation maximization (OSEM) algorithm with scattering, attenuation, and decay corrections applied. Three-dimensional regions of interest (ROIs) were drawn over tumors and major organs, and the radioactivities in the regions were measured and quantified as the percentage injected dose per gram (% ID/g).

For ex vivo biodistribution, mice bearing bilateral tumors (*n* = 5/group) were intravenously injected with 3.7 MBq ^68^Ga-NODAGA-BMS986192. At 1 h and 2 h post-injection, tumor, blood, and selected organs were collected, washed, and weighed, their radioactivities measured on a gamma counter. The counts per minute (CPM) values for each sample were converted to percent of injected dose per gram of tissue (% ID/g). Data were decay-corrected to injection time and shown as means ± standard deviation (SD). To further improve the rigor of our study, a supplementary biodistribution study of blocking (*n* = 3/group) was performed by co-administration of unlabeled ADX_5322_A02 (10 mg/kg) with 3.7 MBq ^68^Ga-NODAGA-BMS986192, tissues were collected at 1 h post-injection.

### PET imaging and radiation dosimetry in cynomolgus

Cynomolgus were first sedated by intramuscular injection of atropine and Shumianling II, and then anesthetized with a mixture of isoflurane and oxygen through endotracheal intubation. Vital parameters were monitored using a portable electrocardiograph (COMEN STAR8000, China). ^68^Ga-NODAGA-BMS986192 at a dose of 5.6 MBq (~ 150 μci)/kg was injected via the cephalic vein. Dynamic images were collected from the calvarium to the hypogastrium (A Field of View = 484 mm) for two hours, and reconstructed in 58 time frames (30 × 2 s, 10 × 1 min, 10 × 5 min, 6 × 10 min). Three-dimensional ROIs were drawn on major organs and the mean standardized uptake values (SUVmean) were computed to form time-activity curves (TACs). Serial whole-body images were acquired by eight consecutive PET scans (15 min per scan) using a continuous bed motion method (3 individual bed positions, 5 min per position), for up to 120 min post-probe injection.

Data for radiation dosimetry was acquired from whole-body ^68^Ga-NODAGA-BMS986192 scans at 10, 25, 40, 70, and 115 min after probe injection, respectively. Source organs were chosen based on the highest probe uptake and previously published reports, which consisted of kidneys, bladder, heart, liver, spleen, adrenals, and the remainder of the body [26]. ROIs over the source organs were contoured manually at the 10-min time point and propagated to later scans based on automatic deformable registration between consecutive scans. OLINDA/EXM software, version 2.1 was used to plot and integrate the kinetic organ activity data for the calculation of total-body and organ time-integrated activity coefficients, residence times, and organ-absorbed doses. Effective doses were calculated using radiation weighting factors from the International Commission on Radiological Protection publication 60 [27].

### Immunohistochemical staining

Harvested tumors (*n* = 3) were fixed, embedded in paraffin, and cut into a 4 μm thick sections. The sections were then deparaffinized to retrieve antigen using 10 mM citrate buffer (pH 6.0). Slides were treated with 3% H_2_O_2_ for 10 min, blocked with 5% goat serum for 1 h, and then incubated with anti-hPD-L1 mouse monoclonal antibody MIH1 (Catalog Number: 14-5983-80) or anti-mPD-L1 rat monoclonal antibody MIH5 (Catalog Number: 14-5982-81) (ThermoFisher, Shanghai, China) at 4 °C overnight. Subsequently, slides were washed, incubated with biotinylated anti-mouse IgG, and visualized by adding DAB chromogen. Sections were counterstained with hematoxylin.

### Biosafety evaluation

Toxicity tests were performed in accordance with the Organization of Economic Co-operation and Development (OECD) guidelines for testing chemicals with minor modifications. A total of 12 healthy C57BL/6 mice (16–18 g, 5–6 weeks old females) were randomly assigned into saline control, acute toxicity (24 h post-injection), and subchronic toxicity (7 d post-injection), in which the treated groups were injected with a single dose (37 MBq) of ^68^Ga-NODAGA-BMS986192. Mice were observed daily for physical appearance (skin, hair, eye, weight), behavior patterns (moving, eating, and sleeping), and signs of injury, pain, and illness. Blood samples were collected for routine blood test as well as cardiac, hepatic, and renal functions. Heart, liver, spleen, lung, and kidney organs were also collected for histopathological examination.

### Statistical analysis

All data were presented as mean ± standard deviation from three to five independent replicates. Statistical analyses were performed using GraphPad Prism 5.0 (GraphPad Software, San Diego, CA, USA), and a *p* value < 0.05 was considered to be statistically significant. Differences between groups were evaluated using Student's *t* test or one-way analysis of variance followed by post hoc Tukey multiple comparisons.

## Results

### Characterization and in vitro stability of.^68^Ga-NODAGA-BMS986192

The molecular weight of the obtained ADX_5322_A02, NODAGA-BMS986192, and the standard reference complex ^69^Ga-NODAGA-BMS986192 were 11162.168, 14212.190, and 14273.838, respectively, as characterized by mass spectroscopy (Additional file 1: Fig. S4). ^68^Ga-NODAGA-BMS986192 was produced with a radiochemistry yield of > 95% (Additional file 1: Fig. S5) and a radiochemistry purity > 99% (Additional file 1: Fig. 6A). The molar activity of the obtained probe was 39.25 ± 2.57 GBq/μmol. It remained stable after incubation in human serum for 2 h at 37℃, with a radiochemical purity of > 99% (Additional file 1: Fig. 6B).

### In vitro binding assay of ^68^Ga-NODAGA-BMS986192 to PD-L1

**Fig. 2:**
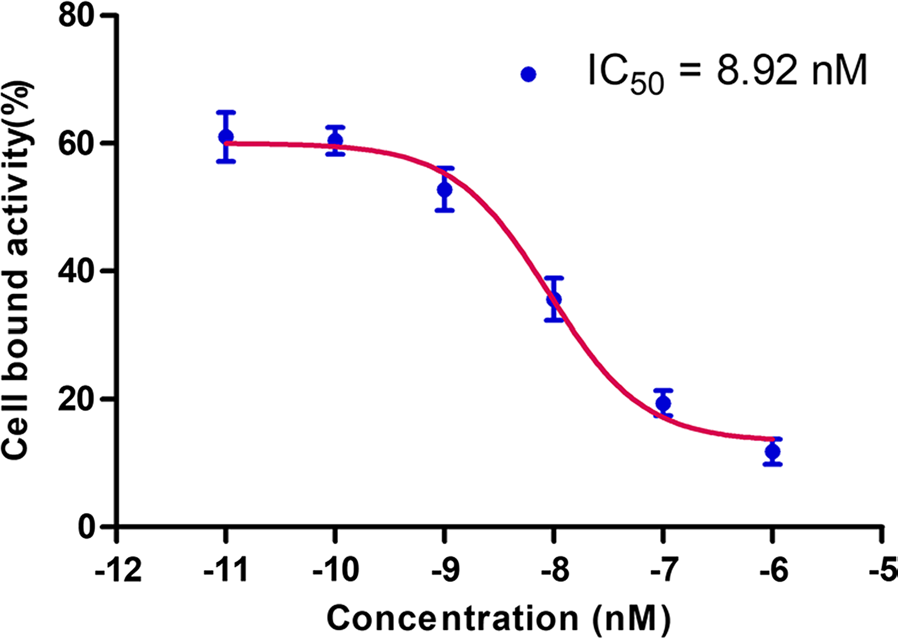
^68^Ga-NODAGA-BMS986192 binding affinity in LN-229 (IC_50_ = 8.92 ± 0.90 nM)

As shown in Fig. 2, ^68^Ga-NODAGA-BMS986192 exhibited a high affinity toward hPD-L1 with an IC_50_ value of 8.92 ± 0.90 nM.

### PET imaging and biodistribution in immunocompetent tumor-bearing mice model

**Fig. 3:**
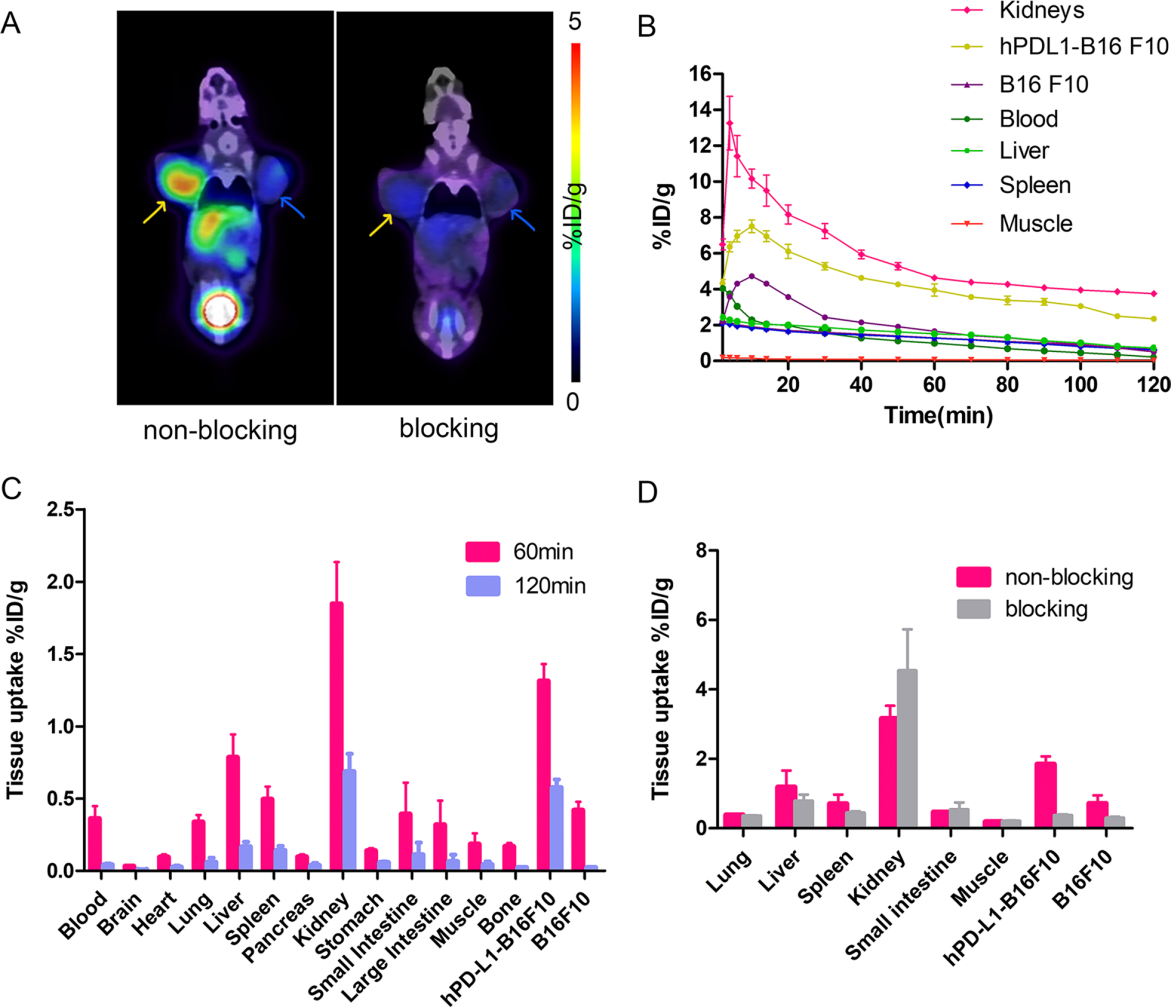
**A** Representative coronal slices PET images of two mice bearing bilateral hPD-L1^+^ hPD-L1-B16F10 (yellow arrows) and hPD-L1^−^ B16F10 (blue arrows) tumors at 60 min post-^68^Ga-NODAGA-BMS986192 administration. The left images show probe alone; the right images show co-administration of 10 mg/kg ADX_5322_A02; **B** Representative time-activity curves; **C** Mice ex vivo biodistribution of ^68^Ga-NODAGA-BMS986192, 1 h and 2 h post-injection (*n* = 5/group); **D** Mice ex vivo biodistribution of.^68^Ga-NODAGA-BMS986192 1 h post-injection, with and without co-administration of cold ADX_5322_A02 as a blocking agent (*n* = 3/group)

Coronal PET images of bilateral tumor models are shown in Fig. 3A. The ^68^Ga-NODAGA-BMS986192 probe accumulated rapidly in the hPD-L1-B16F10 tumor, and produced satisfactory images at 1 h post-injection. In contrast, probe accumulation in the wide-type B16F10 tumor was significantly lower than that in hPD-L1-B16F10 (1.03 ± 0.16 vs. 3.54 ± 0.35%ID/g, *p* < 0.05). Blocking with an excess of unlabeled ADX_5322_A02 (10 mg/kg), on the other hand, reduced the probe uptake by 65 ± 7% in the hPD-L1-B16F10 tumors, confirming the target specificity of the probe. There was a considerable variation in the peak radioactivity within the tumor microenvironment, as detected by TACs of PET imaging. This variation can be attributed to the extent of tumor blood flow and the massive excretion from the kidneys. Nevertheless, the tumor uptake of radioactivity decreased gradually over the first hour post-injection, and then slowly plateaued for the later time points. In comparison, the muscular uptake of radioactivity increased from 0 ~ 5 min post-injection, and then decreased steadily, yielding better contrast for images at later time points (Fig. 3B).

Ex vivo biodistribution results were consistent with the PET images (Fig. 3C). The high uptake of the probe in kidney underpinned its important role in probe clearance. The probe was mostly cleared from the blood pool within two hours. The tumor-to-muscle ratios of probe uptake in the hPD-L1-B16F10 group at 1 and 2 h post-injection were 7.28 ± 0.63 and 9.15 ± 1.10, respectively. Blocking with an excessive unlabeled ADX_5322_A02, similar to the imaging findings, reduced the probe uptake by 74 ± 12% in the hPD-L1-B16F10 tumors. The blocking also caused slight reduction of probe accumulation in the wild-type B16F10 tumor, liver, and spleen (0.65 ± 0.11 vs 0.29 ± 0.04%ID/g, 1.07 ± 0.37 vs 0.71 ± 0.20%ID/g, 0.64 ± 0.08 vs 0.43 ± 0.06%ID/g), respectively (Fig. 3D).

### Cynomolgus imaging and radiation dosimetry

**Fig. 4:**
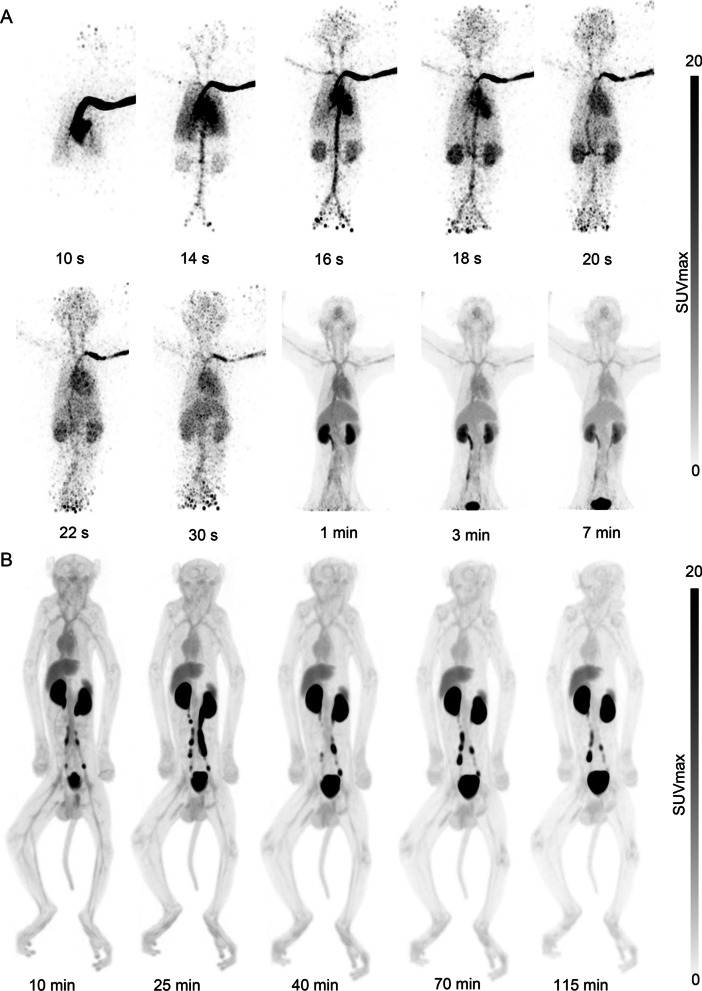
Cynomolgus MIP images of.^68^Ga-NODAGA-BMS986192. **A** Representative dynamic images; **B** Serials whole-body static images

**Fig. 5:**
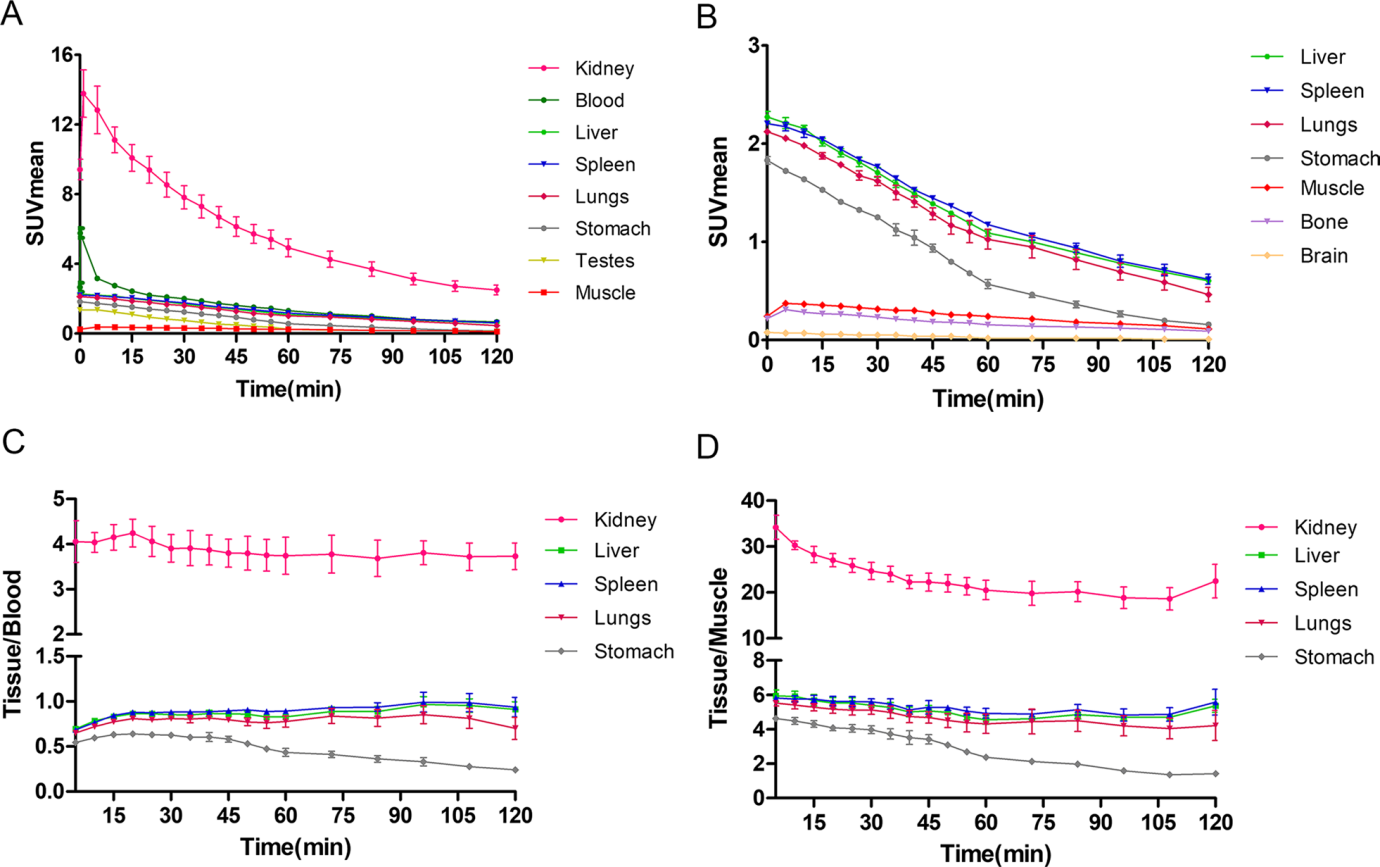
**A, B** Cynomolgus major organs dynamic time-activity curves; **C**, **D** Major organs tissue-to-blood and tissue-to-muscle ratios with time

The dynamic and serial whole-body images of the cynomolgus were presented as maximum intensity projections (MIPs) (Fig. 4). PET ROI analyses were shown as time-activity curves (Fig. 5A, B). Figure 5A reveals that renal excretion was the main route of clearing the ^68^Ga-NODAGA-BMS986192, and the blood pool radioactivity decreased by 50% within 20 min post-injection. The SUVmean in the spleen and liver at 60 min post-injection was 1.59 ± 0.18 and 1.21 ± 0.13, respectively. The spleen uptake can be attributed to the interaction of the probe with PD-L1 on lymphocytes and dendritic cells, while the liver uptake was probably caused by catabolism of the probe [19]. The accumulation of radioactivity in the kidneys, liver, and spleen diminished over time, yet was visible in the PET images throughout the experiment. The probe accumulation in muscle and bone was minimal, consistent with low background radioactivity and high-contrast images. The optimal time window for PET imaging was 45 to 75 min p.i, as determined by the changes in major organs tissue-to-blood and tissue-to-muscle ratios over time (Fig. 5C, D).

Table 1 summarizes the pooled cynomolgus dosimetry reports from OLINDA/EXM software, version 2.1. Organs with the highest effective doses were the kidney (1.47E−03 mSv/MBq), followed by the thyroid (7.03E−04 mSv/MBq), liver (6.94E−04 mSv/MBq), and thymus (6.26E−04 mSv/MBq). The total-body effective dose was 6.34E−03 mSv/MBq. It should be noted that the reported standard deviations were calculated from the mean dosimetry profiles of the 3 male cynomolgus, and therefore did not account for any possible errors in organ delineation.

**Table 1:** ^68^Ga-NODAGA-BMS986192 Dosimetry Summary of Effective Doses in healthy male cynomolgus (mSv/MBq)

Organs	Monkey1	Monkey2	Monkey3	Average	SD
Adrenals	5.75E−04	6.41E−04	4.33E−04	5.50E−04	1.06E−04
Brain	3.25E−05	3.24E−05	2.90E−05	3.13E−05	1.99E−06
Cortical Bone	3.64E−06	5.69E−06	6.67E−06	5.33E−06	1.55E−06
Esophagus	3.25E−04	3.67E−04	3.09E−04	3.34E−04	3.00E−05
Eyes	0.00E+00	0.00E+00	0.00E+00	0.00E+00	0.00E+00
Gallbladder Wall	3.56E−04	2.34E−05	2.66E−04	2.15E−04	1.72E−04
Heart Contents	4.89E−05	2.21E−05	3.33E−05	3.48E−05	1.35E−05
Heart Wall	7.66E−06	4.32E−05	6.52E−05	3.87E−05	2.90E−05
Kidneys	1.03E−03	2.21E−03	1.16E−03	1.47E−03	6.47E−04
Liver	9.41E−04	7.31E−04	4.09E−04	6.94E−04	2.68E−04
Lungs	7.06E−05	5.06E−05	3.20E−05	5.11E−05	1.93E−05
Left colon	4.33E−04	3.98E−05	4.27E−04	3.00E−04	2.25E−04
Muscle	6.78E−06	3.21E−06	4.42E−06	4.80E−06	1.82E−06
Osteogenic Cells	3.22E−06	3.89E−06	2.27E−06	3.13E−06	8.14E−07
Pancreas	2.25E−04	2.37E−04	2.94E−04	2.52E−04	3.69E−05
Prostate	6.03E−05	7.99E−06	4.06E−05	3.63E−05	2.64E−05
Rectum	6.02E−05	4.43E−06	4.29E−05	3.58E−05	2.85E−05
Red Marrow	4.33E−06	7.29E−07	5.50E−07	1.87E−06	2.13E−06
Salivary Glands	3.02E−04	5.88E−05	3.23E−05	1.31E−04	1.49E−04
Small Intestine	4.23E−04	4.88E−05	4.54E−04	3.09E−04	2.26E−04
Spleen	1.75E−04	1.05E−04	5.44E−05	1.11E−04	6.06E−05
Stomach Contents	5.01E−05	5.87E−05	5.48E−05	5.45E−05	4.31E−06
Stomach Wall	3.67E−04	3.07E−05	4.01E−04	2.66E−04	2.05E−04
Testes	5.88E−05	5.45E−05	3.22E−05	4.85E−05	1.43E−05
Thymus	6.43E−04	5.55E−04	6.79E−04	6.26E−04	6.38E−05
Thyroid	8.43E−04	7.40E−04	5.25E−04	7.03E−04	1.62E−04
Urinary Bladder Contents	2.12E−05	1.13E−05	2.11E−05	1.79E−05	5.69E−06
Urinary Bladder Wall	2.35E−05	1.27E−05	2.20E−05	1.94E−05	5.85E−06
Total Body	7.09E−03	6.10E−03	5.83E−03	6.34E−03	6.63E−04

## Immunohistochemistry

**Fig. 6:**
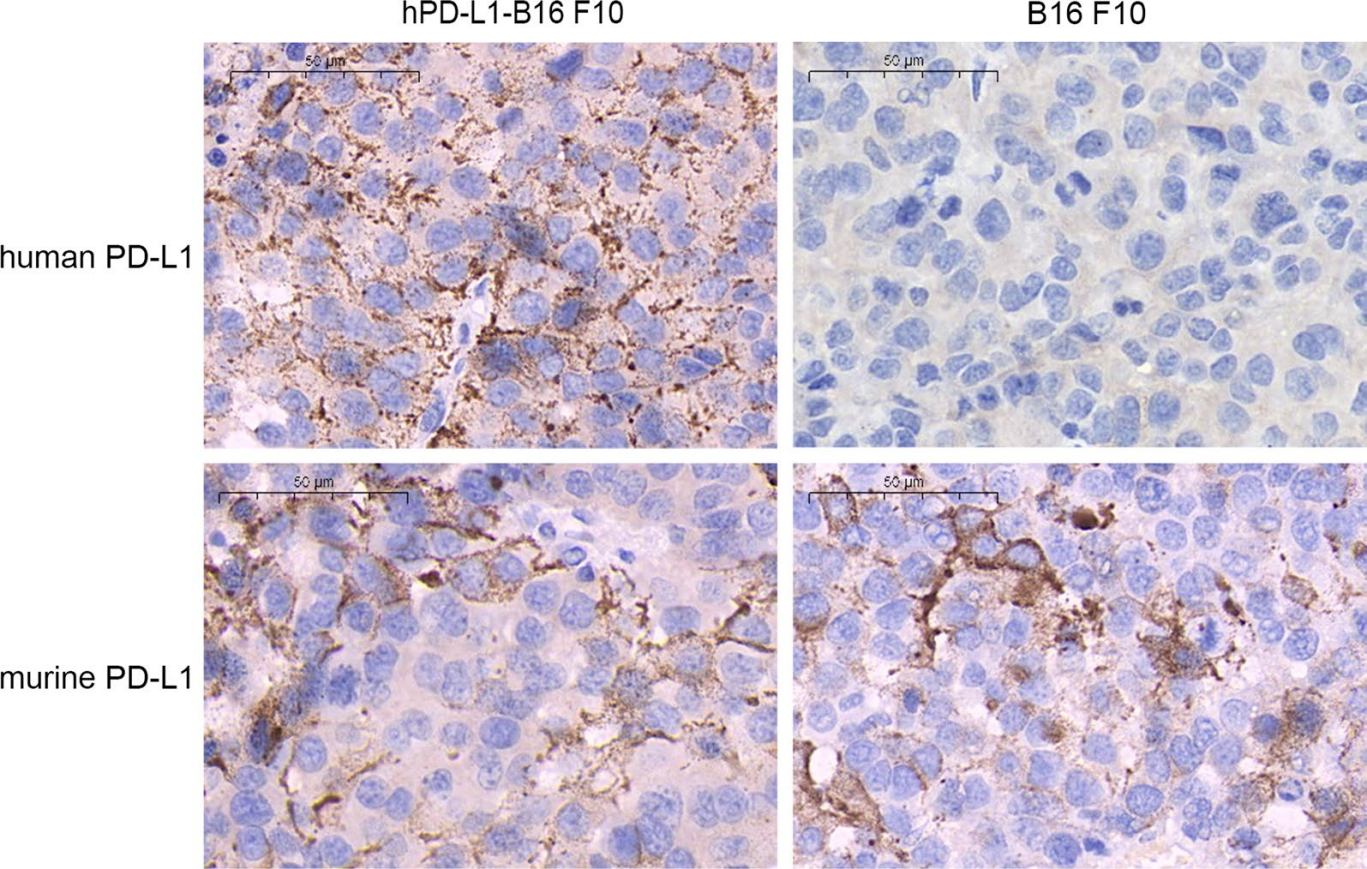
Representative images of hPD-L1 expression in the hPD-L1-B16F10 tumors (400, Bar = 50 μm)

Murine PD-L1 was expressed in about 20% of the B16F10 cells regardless of transfection (Fig. 6). On the other hand, human PD-L1 was stably expressed in more than 90% of the B16F10 after transfection, with a membranous staining pattern.

## Biosafety evaluation

None of the treated mice exhibited any signs of anomaly in terms of physical appearance or behavior patterns. There was no significant difference in body weight between the control and the subchronic toxicity group at the end of the observation (Additional file 1: Fig. S7). The treated groups had similar results from the routine blood test to that of the control, including creatine kinase (CK), alanine aminotransferase (ALT), aspartate aminotransferase (AST), and urea nitrogen (UREA). [28, 29] (Additional file 1: Tables S1 & S2). Pathological examination also revealed no significant alterations, including organ damage, inflammation, and necrosis, in the treated mice (Additional file 1: Fig. S8).

## Discussion

This study reported an optimized ^68^Ga-NODAGA-BMS986192 by replacing the bifunctional chelator DOTA with NODAGA. The thermodynamic stability constant of ^68^Ga-NODAGA (log K = 30.98) is much higher than that of ^68^Ga-DOTA (log K = 21.33). The coordination cavity of DOTA, consisted of four nitrogen atoms and four oxygen atoms, is too big for ^68^Ga^3+^, whereas only four nitrogen atoms and two oxygen atoms contribute to the actual chelation. In comparison, the coordination cavity of NODAGA chelates ^68^Ga^3+^ with a higher stability [30–32]. Compared to the published research [21], the radiolabeling procedure of NODAGA-BMS986192 required lower temperature and shorter reaction time. The resultant product exhibited an outstanding stability in human serum after 2 h of incubation (RCP > 99% vs. RCP > 97%) and a similar binding affinity to hPD-L1 (8.92 ± 0.90 nM vs. 2.0 ± 0.6 nM) as compared to the published counterparts. Therefore, we conclude that substitution of the chelating module did not compromise the binding affinity of this PET probe to human PD-L1.

The PET imaging and ex vivo biodistribution studies demonstrated the rapid and homing of the radioprobe toward hPD-L1 positive tumors (Fig. 3). The blocking test also verified the specificity of ^68^Ga-NODAGA-BMS986192 toward hPD-L1. In comparison, no blocking effect was observed in the B16-F10 wild-type tumors (murine PD-L1 positive) and other immune organs [33], confirming the low affinity of ^68^Ga-NODAGA-BMS986192 toward murine PD-L1 or non-specific accumulation.

The PET images and TACs of NHPs (Figs. 4, 5) showed a rapid probe clearance through renal and negligent retention in most non-target tissues and the optimal time window to be 45 to 75 min p.i. These results were in line with the satisfactory target-to-background ratio for the ^68^Ga-NODAGA-BMS986192 to assess tumor PD-L1 expression and stratify patients that can potentially benefit from anti-PD-L1 treatment. In addition, no excess unlabeled precursor was needed during PET probe administration due to the low overall background, which result in a feasible and costless medical examination than antibodies [20]. Notably, according to the dosimetry analysis in cynomolgus, the estimated total-body effective dose was 6.34E−03 mSv/MBq, which was lower than that of ^68^Ga-FAPI-46 (7.80E−03 mSv/MBq) [34] or ^68^Ga-PSMA-11 (1.08E−02 mSv/MBq) [35]. Together with the results of biosafety evaluation, nearly no detrimental effects caused by the ^68^Ga-NODAGA-BMS986192 to major organs. Therefore, this probe may have a better safety profile for further clinical translation. Moreover, the excretion of radioprobe from patients can be accelerated by drinking and urination which can further improve imaging quality and reduce radiation exposure.

Compared to reported preclinical studies based on ADX_5322_A02 [18, 21], all the probes showed high specificity for hPD-L1 detection and demonstrated similar pharmacokinetics with fast renal clearance. In this study, we optimized the ^68^Ga labeling method and obtained ^68^Ga-labeled BMS-986192 with higher stability. However, the uptake of the radiotracer in the hPD-L1^+^ tumor was lower than that in the previous reports, probably because the murine PD-L1 expressed in wild-type B16F10, albeit with low affinity, still trapped some of the tracers from the blood circulation. Immunocompetent murine models have been widely used in Immuno-SPECT/PET imaging [10, 25, 33, 36]. And it was recorded that immune-associated organs and additional PD-L1-rich organs can impact the distribution of the anti-PD-L1 PET probe [24]. Therefore, the immunocompetent mice might be more representable for the metabolism of ^68^Ga-NODAGA-BMS986192 compared to immune-deficient Balb/c and NSG mice. Furthermore, radiation dosimetry analysis in NHPs provided vital information to make clinical transitions of ^68^Ga-NODAGA-BMS986192 move forward.

In brief, the Adnectin-based PET probe, ^68^Ga-NODAGA-BMS986192, has shown very promising results as imaging agents for their favorable physicochemical properties, such as small size, high stability, high affinity and specificity, rapid tumor uptake, and normal tissue clearance. Moreover, the mild radiolabeling conditions and rapid imaging after 1 h p.i as well as the outstanding biosafety and radio-safety profile make it potential for clinical application of PD-L1 imaging. Collectively, these results are highly encouraging for the application of ^68^Ga-NODAGA-BMS986192 to screen the beneficiaries of immunotherapy and predict the efficacy of anti-PD-L1 treatment in clinical application.

Our study has some limitations. First, PET imaging of hPD-L1 was performed in the hPD-L1-B16F10 bearing C57BL/6 mice model instead of a transgenic mice model with humanized PD-L1 [37]. Although our hPD-L1 expression mice models confirmed hPD-L1 visualization with PET probe, a transgenic mice model with human PD-L1 expression would provide a more accurate assessment of probe accumulation in immune-associated organs. Second, because of the limited access to small animal PET, all scans were performed with an uBioEXPLORER PET/CT scanner which yields a 374 (transaxial) 484 (axial) mm^2^ field of view. Although this scanner provided an excellent total-body imaging of large animals, the image resolution was sub-optimal for tumor-bearing mice. Third, only male cynomolguses were enrolled for the radiation dosimetry study, which may lead to gender bias, and cautions should be taken when extrapolating the results. Fourth, a second tumor model with endogenously low expression of PD-L1 would help further validate the performance of this probe.

## Conclusion

We developed an optimized Adnectin-based anti-hPD-L1 probe (^68^Ga-NODAGA-BMS986192) offering hPD-L1 visualization in moderate and flexible radiolabeling conditions. Evaluation of the developed anti-hPD-L1 radioprobe in immune-activity mice provided effective scrutiny of the interaction between radioprobe and the immune microenvironment. Coupling with the dosimetry assay in NHPs with the advantage of similarities to human beings, our findings could facilitate the transition of ^68^Ga-NODAGA-BMS986192 for clinical PET imaging of hPD-L1.

## Supplementary Information

**Additional file 1**. **Figure S1** (**A**) The 10th type III domain of human fibronectin, BC, DE, and FG loops represent variable domains; (**B**) The core sequence of ADX_5322_A02 (underlined part representing the binding site of hPD-L1). **Figure S2** (**A**) DNA sequence report of the ADX_5322_A02; (**B, C**) SDS–PAGE results of Adnectin expression and purification. **Figure S3** hPD-L1-B16F10 cell line RT-qPCR results indicated that the human CD274 RNA expression rate is 556047% overexpressed compared to B16F10 wild type. **Figure S4** (**A, B, C** in turn) The accurate molecular weight of obtained ADX_5322_A02, NODAGA-Adnectin, and the standard reference ^69^ Ga-NODAGA-BMS986192 determined by MALDI-TOF mass spectrometry. **Figure S5** Representative iTLC result of ^68^Ga-NODAGA-BMS986192. **Figure S6** (**A**) HPLC analysis of ^68^Ga-NODAGA-BMS986192. The UV-chromatogram retention time was 11.627 min, and the radio-chromatogram 12.071 min; (**B**) In vitro serum incubation of ^68^Ga-NODAGA-BMS986192 up to 2 h determined by Radio-HPLC demonstrated a satisfactory stability of RCP > 99%. **Figure S7** The growth curves of mice (n = 4). Subchronic group (37 MBq ^68^Ga-NODAGA-BMS986192), control group (saline). **Figure S8** Representative images of H&E eosin staining (100, Bar = 100 μm). Neither noticeable organ impairment nor obvious inflammation or necrosis was observed for all groups. **Table S1** The results of blood routine test (n = 4). **Table S2** The results of biochemical analyses (n = 4).

## Data Availability

All data generated or analyzed during this study are included in this published article and its supplementary information files.

## Abbreviations

ALT: Alanine aminotransferase
AST: Aspartate aminotransferase
BFC: Bifunctional chelator
CK: Creatine kinase
CPM: Counts per minute
DMEM: Dulbecco's Modified Eagle Medium (DMEM)
DMSO: Dimethyl sulfoxide
DOTA: 1,4,7,10-Tetraazacyclododecane-*N*,*N*',*N,N'*-tetraacetic acid
HPLC: High-performance liquid chromatography
hPD-L1: Human PD-L1
hPD-L1-B16F10: HPD-L1 gene transfected B16-F10
ICIs: Immune checkpoint inhibitors
IHC: Immunohistochemical
iTLC: Instant thin-layer chromatography
mAbs: Monoclonal antibodies
MALDI-TOF: Matrix-assisted laser desorption/ionization time-of-flight
MIPs: Maximum intensity projections
mPD-L1: Murine PD-L1
NHPs: Non-human primates
NODAGA: 1,4,7-Triazacyclononane-*N*-glutamic acid-*N*′,*N*″-diacetic acid
NSCLC: Non-small cell lung carcinoma
OSEM: Ordered subsets expectation maximization
PET: Positron emission tomography
PD-L1: Programmed death ligand-1
RCY: Radiochemical yield
RCP: Radiochemical purity
ROIs: Regions of interest
SUVmax/mean: Max/mean standardized uptake values
TACs: Time activity curves
TCEP: Tris(2-chloroethyl) phosphate
^10^Fn3: The 10th type III domain of human fibronectin
%ID/g: Percentage injected dose per gram
Urea: Urea nitrogen
