# Supplementary material for: PET imaging of an optimized anti-PD-L1 probe 68Ga-NODAGA-BMS986192 in immunocompetent mice and non-human primates
Source: EJNMMI Res. 2022 Jun 13;12:35. doi: 10.1186/s13550-022-00906-x (PMC9192916; doi:10.1186/s13550-022-00906-x)
Supplement: Supplementary file 1 — Additional file 1. Figure S1 (A) The 10th type III domain of human fibronectin, BC, DE, and FG loops represent variable domains; (B) The core sequence of ADX_5322_A02 (underlined part representing the binding site of hPD-L1). Figure S2 (A) DNA sequence report of the ADX_5322_A02; (B, C) SDS–PAGE results of Adnectin expression and purification. Figure S3 hPD-L1-B16F10 cell line RT-qPCR results indicated that the human CD274 RNA expression rate is 556047% overexpressed compared to B16F10 wild type. Figure S4 (A, B, C in turn) The accurate molecular weight of obtained ADX_5322_A02, NODAGA-Adnectin, and the standard reference 69 Ga-NODAGA-BMS986192 determined by MALDI-TOF mass spectrometry. Figure S5 Representative iTLC result of 68Ga-NODAGA-BMS986192. Figure S6 (A) HPLC analysis of 68Ga-NODAGA-BMS986192. The UV-chromatogram retention time was 11.627 min, and the radio-chromatogram 12.071 min; (B) In vitro serum incubation of 68Ga-NODAGA-BMS986192 up to 2 h determined by Radio-HPLC demonstrated a satisfactory stability of RCP > 99%. Figure S7 The growth curves of mice (n = 4). Subchronic group (37 MBq 68Ga-NODAGA-BMS986192), control group (saline). Figure S8 Representative images of H&E eosin staining (100, Bar = 100 μm). Neither noticeable organ impairment nor obvious inflammation or necrosis was observed for all groups. Table S1 The results of blood routine test (n = 4). Table S2 The results of biochemical analyses (n = 4). [file 13550_2022_906_MOESM1_ESM.docx]

**Additional Files**

**PET imaging of an optimized anti-PD-L1 probe** **^68^Ga-NODAGA-BMS986192 in immunocompetent mice and non-human primates**

**Running title:** ^68^Ga-NODAGA-BMS986192 for PET imaging

**Authors:**

Huimin Zhou^1^, Guangfa Bao^1^, Ziqiang Wang^1^, Buchuan Zhang^1^, Dan Li^1^, Lixing Chen^1^, Xiaoyun Deng^1^, Bo Yu^1^, Jun Zhao^1,2,3^, Xiaohua Zhu^1,^*

**Affiliation:**

^1^Department of Nuclear Medicine, Tongji Hospital, Tongji Medical College, Huazhong University of Science and Technology, Wuhan 430030, China

^2^Department of Anatomy, School of Basic Medicine, Huazhong University of Science and Technology, Wuhan, Hubei Province 430030, China

^3^Cell Architecture Research Center, Huazhong University of Science and Technology, Wuhan, Hubei, 430030, China

***Corresponding Author:**

Xiaohua Zhu, MD, PhD. Department of Nuclear Medicine, Tongji Hospital, Tongji Medical College, Huazhong University of Science and Technology. 1095 Jiefang Ave, Wuhan 430030, China. Email: [evazhu@vip.sina.com](mailto:evazhu@vip.sina.com). Tel: +86-27-83663446. Fax: +86-27-83663446. ORCID：0000-0003-0495-9510

**
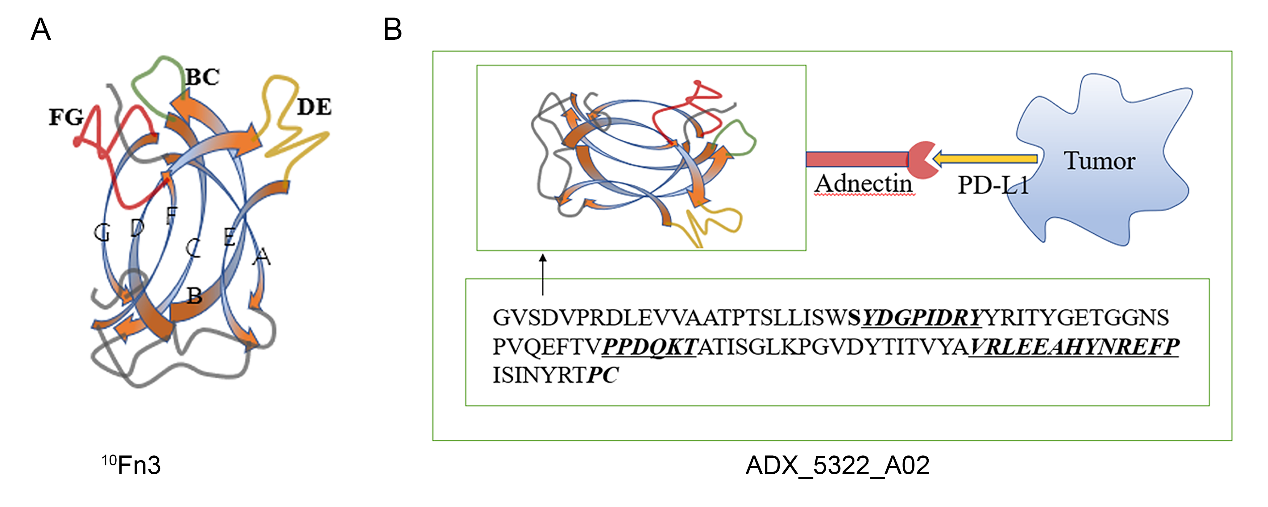
**

**Supplemental Figure 1.** (A) The 10^th^ type III domain of human fibronectin, BC, DE, and FG loops represent variable domains; (B) The core sequence of ADX_5322_A02 (underlined part representing the binding site of hPD-L1)


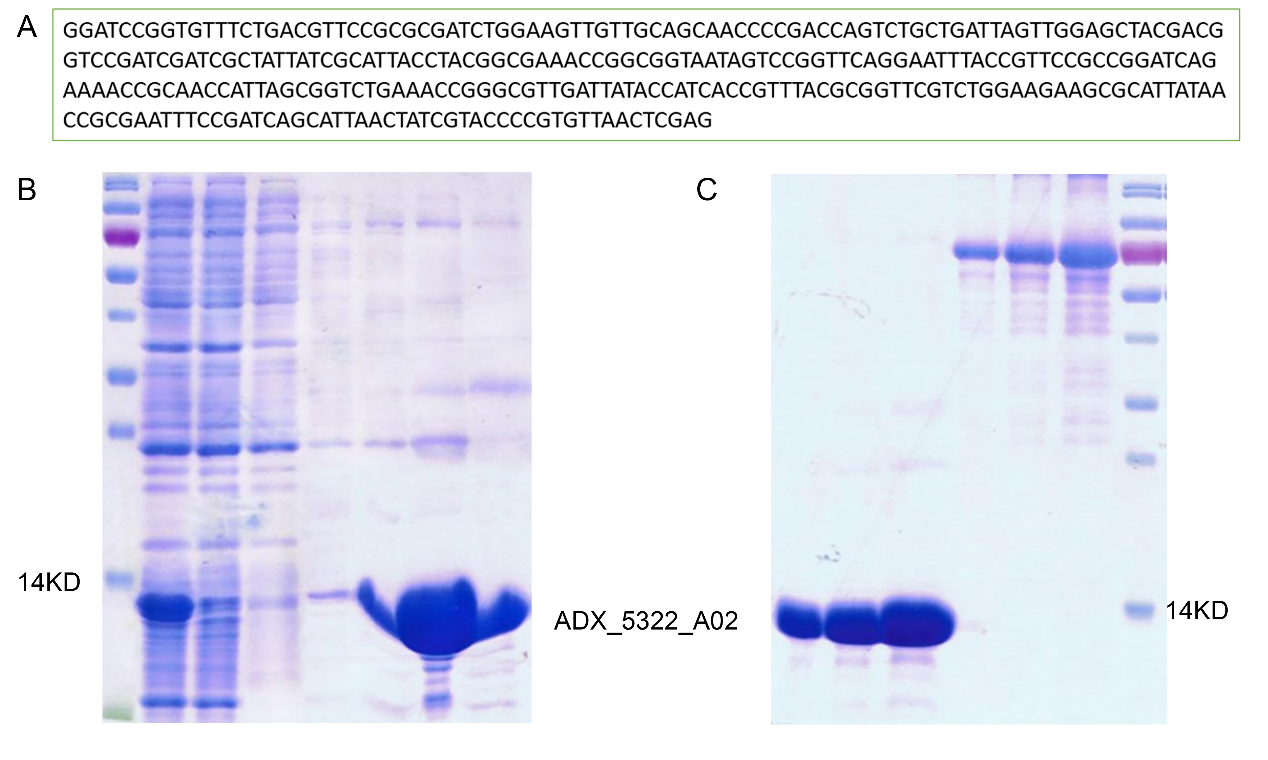


**Supplemental Fig**ure **2**. (A) DNA sequence report of the ADX_5322_A02; (B, C) SDS-PAGE results of Adnectin expression and purification


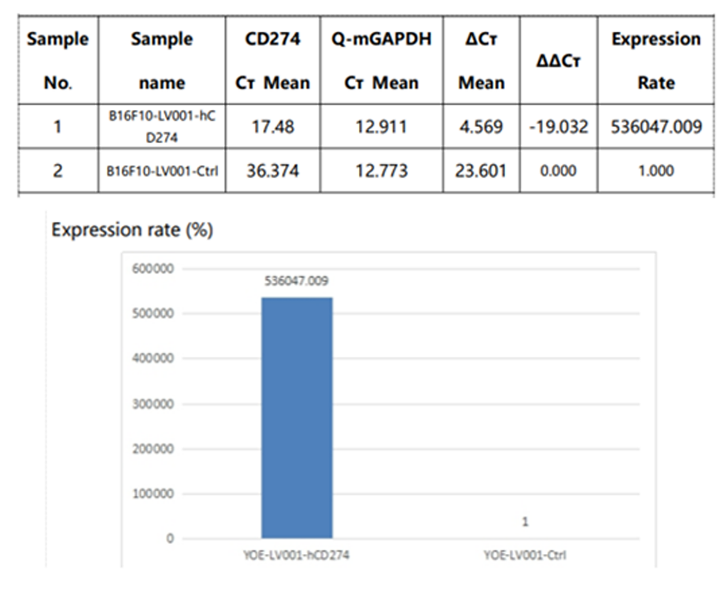


**Supplemental Figure** **3.** hPD-L1-B16F10 cell line RT-qPCR results indicated that the human CD274 RNA expression rate is 556047% overexpressed compared to B16F10 wildtype

**
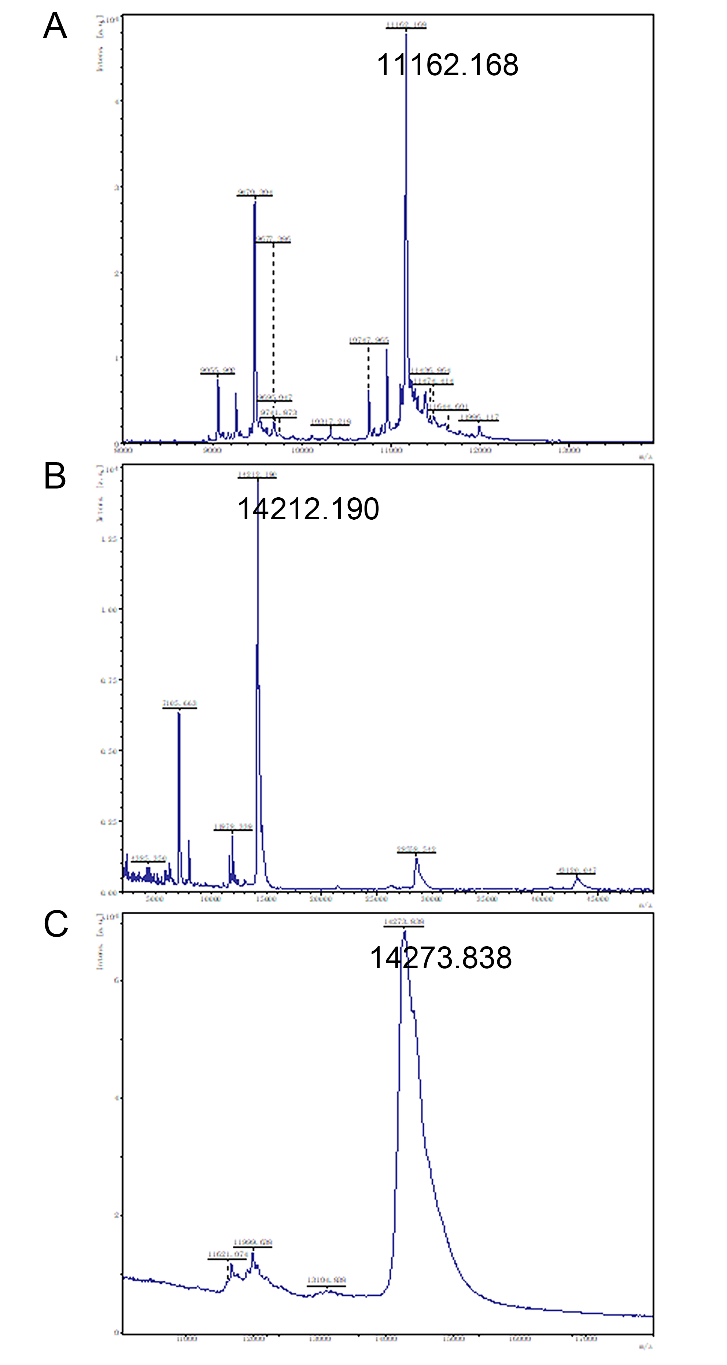
**

**Supplemental Figure** **4.** (A, B, C in turn) The accurate molecular weight of obtained ADX_5322_A02, NODAGA-Adnectin, and the standard reference ^69^Ga-NODAGA-BMS986192 determined by MALDI-TOF mass spectrometry


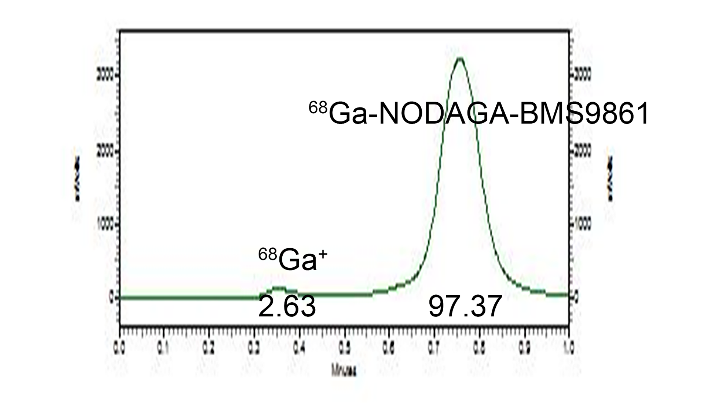


**Supplemental Figure 5.** Representative iTLC result of ^68^Ga-NODAGA-BMS986192


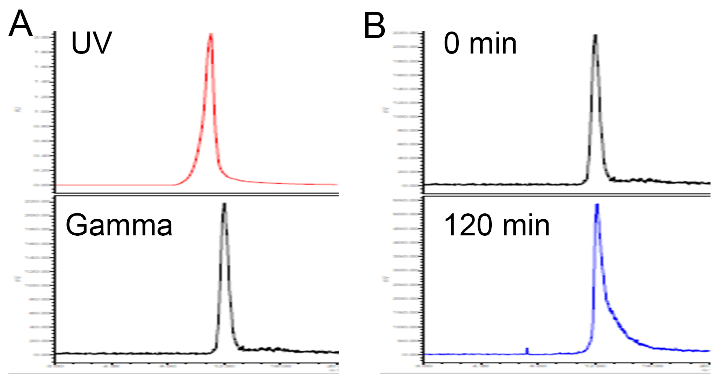


**Supplemental Figure** **6.** (A) HPLC analysis of ^68^Ga-NODAGA-BMS986192. The UV-chromatogram retention time was 11.627 min, and the radio-chromatogram 12.071 min; (B) In vitro serum incubation of ^68^Ga-NODAGA-BMS986192 up to 2 h determined by Radio-HPLC demonstrated a satisfactory stability of RCP > 99%


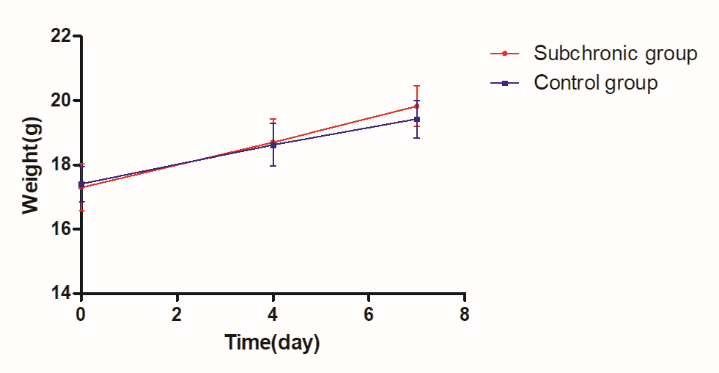


**Supplemental Figure** **7.** The growth curves of mice (n = 4). Subchronic group (37 MBq ^68^Ga-NODAGA-BMS986192), control group (saline)


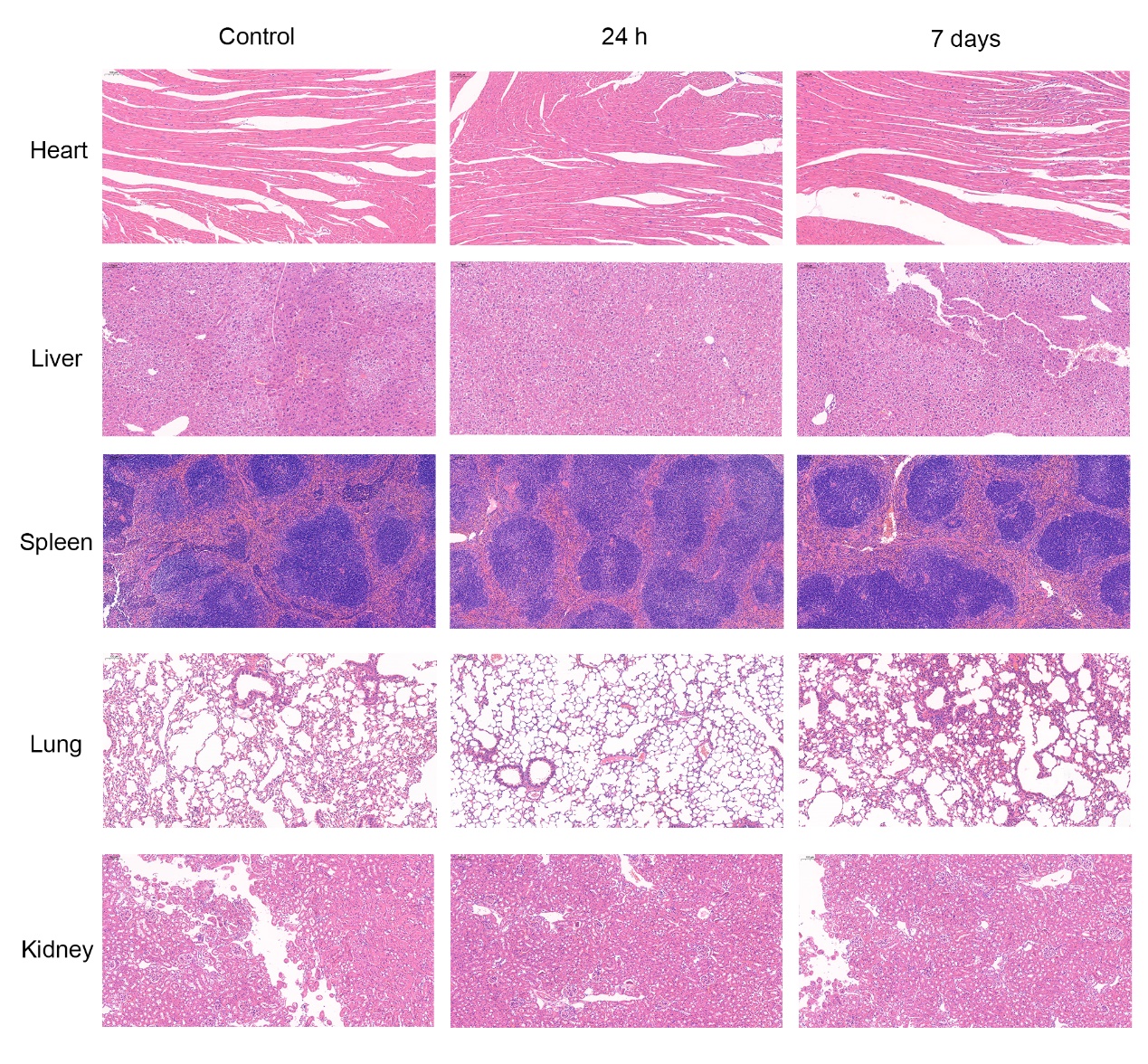


**Supplemental Figure** **8.** Representative images of hematoxylin & eosin staining (🞨100, Bar = 100 μm). Neither noticeable organ impairment nor obvious inflammation or necrosis was observed for all groups

**Supplemental Table** **1.** The results of blood routine test (n = 4)

| Parameters | Control | 24 h | 7 days | Units | Reference Ranges |
| --- | --- | --- | --- | --- | --- |
| WBC | 4.3 ± 1.2 | 3.7 ± 0.9 | 3.4 ± 1.5 | 10^9^/L | 0.8-6.8 |
| Lymph# | 4.1 ± 0.8 | 2.2 ± 0.9 | 3.5 ± 0.5 | 10^9^/L | 0.7-5.7 |
| Mon# | 0.2 ± 0.0 | 0.2 ± 0.0 | 0.2 ± 0.0 | 10^9^/L | 0.0-0.3 |
| Gran# | 1.2 ± 0.2 | 1.3 ± 0.1 | 1.5 ± 0.1 | 10^9^/L | 0.1-1.8 |
| Lymph% | 73.6 ± 3.2 | 64.9 ±6.8 | 70.2 ± 2.7 | % | 55.8-90.6 |
| Mon% | 2.7 ± 0.4 | 5.1 ± 0.3 | 3.5 ± 0.9 | % | 1.8-6.0 |
| Gran% | 23.7 ± 7.2 | 27.9 ± 2.2 | 26.3 ± 4.6 | % | 8.6-38.9 |
| RBC | 8.25 ± 1.11 | 7.04 ±1.01 | 8.45 ± 0.33 | 10^12^/L | 6.36-9.42 |
| HGB | 123 ± 11 | 129 ± 10 | 117 ±3 | g/L | 110-143 |
| HCT | 40.4 ± 2.3 | 37.9 ± 2.9 | 39.7 ± 2.7 | % | 34.6-44.6 |
| MCV | 52.2 ± 1.0 | 55.9 ± 2.5 | 50.0 ± 1.7 | fL | 48.2-58.3 |
| MCH | 17.4 ± 0.7 | 15.9 ± 0.4 | 17.8 ± 0.9 | pg | 15.8-19 |
| MCHC | 312 ± 6 | 318 ± 8 | 304 ± 4 | g/L | 302-353 |
| RDW | 16.3 ± 0.2 | 16.8 ± 0.7 | 15.7 ± 1.1 | % | 13-17 |
| PLT | 1484 ± 113 | 1501 ± 91 | 1365 ± 146 | 10^9^/L | 450-1590 |
| MPV | 5.6 ± 0.3 | 4.6 ± 1.1 | 5.5 ± 0.6 | fL | 3.8-6.0 |

**Supplemental Table** **2.** The results of biochemical analyses (n = 4)

| Parameters | Control | 24 h | 7 days | Reference Ranges |
| --- | --- | --- | --- | --- |
| CK | 605.32 ± 108.22 | 985.77 ± 99.70 | 498.07 ± 302.53 | 0-2070.55 U/L |
| ALT | 38.54 ± 7.99 | 22.17 ± 7.26 | 20.16 ± 9.13 | 10.06-96.47 U/L |
| AST | 133.37 ± 20.76 | 127.84 ±6.28 | 125.57 ± 21.37 | 36.31-235.48 U/L |
| UREA | 8.23 ± 1.33 | 7.27 ± 1.10 | 8.02 ± 1.64 | 5.7-11.7 mmol/L |
